# Supplementary material for: The long and the short of it: unlocking nanopore long-read RNA sequencing data with short-read differential expression analysis tools
Source: NAR Genom Bioinform. 2021 Apr 26;3(2):lqab028. doi: 10.1093/nargab/lqab028 (PMC8074342; doi:10.1093/nargab/lqab028)
Supplement: lqab028_Supplemental_File [file lqab028_supplemental_file.pdf]

Published online *Nucleic Acids Research, 0000, Vol. 00, No. 00* ***1–1***
doi:10.1093/nar/gkn000

Supplementary materials: The long and the short of it: unlocking nanopore long-read RNA sequencing data with short-read differential expression analysis tools

Xueyi Dong[1,2], Luyi Tian[1,2], Quentin Gouil[1,2], Hasaru Kariyawasam[1], Shian Su[1,2], Ricardo De Paoli-Iseppi[3], Yair David Joseph Prawer[3], Michael B. Clark[3], Kelsey Breslin[1], Megan Iminitoff[1,2], Marnie E. Blewitt[1,2], Charity W. Law[1,2], Matthew E. Ritchie[1,2]

[1]Epigenetics and Development Division, The Walter and Eliza Hall Institute of Medical Research, 1G Royal Parade, Parkville, Victoria 3052, Australia and [2]Department of Medical Biology, The University of Melbourne, Parkville, Victoria 3010, Australia and [3]Centre for Stem Cell Systems, Department of Anatomy and Neuroscience, The University of Melbourne, Parkville, Victoria 3010, Australia

© The Author(s)
This is an Open Access article distributed under the terms of the Creative Commons Attribution Non-Commercial License (http://creativecommons.org/licenses/by-nc/2.0/uk/) which permits unrestricted non-commercial use, distribution, and reproduction in any medium, provided the original work is properly cited.

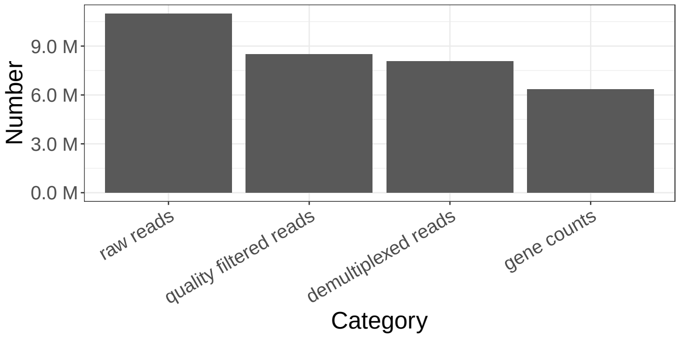

Figure S1. The number of raw reads,quality filtered (average base quality score >7) reads, trimmed and demultiplexed reads and gene-level counts in the sequins long-read dataset.

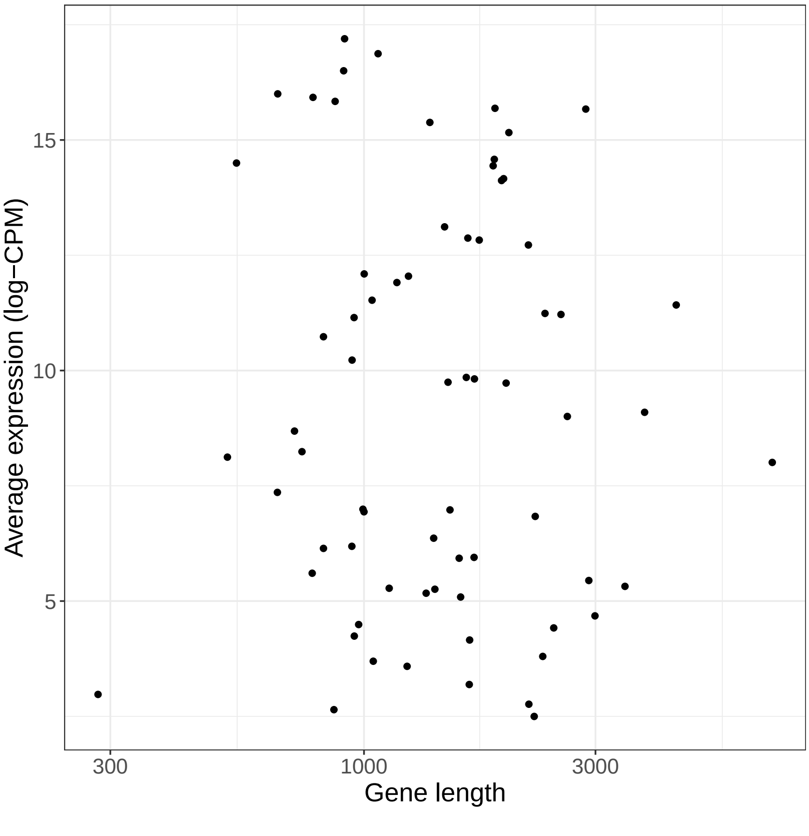

Figure S2. Correlation between gene length and average gene expression (log-CPM) in the sequins dataset. Gene length is weakly associated with expression (Pearson correlation coefficient=-0.056).

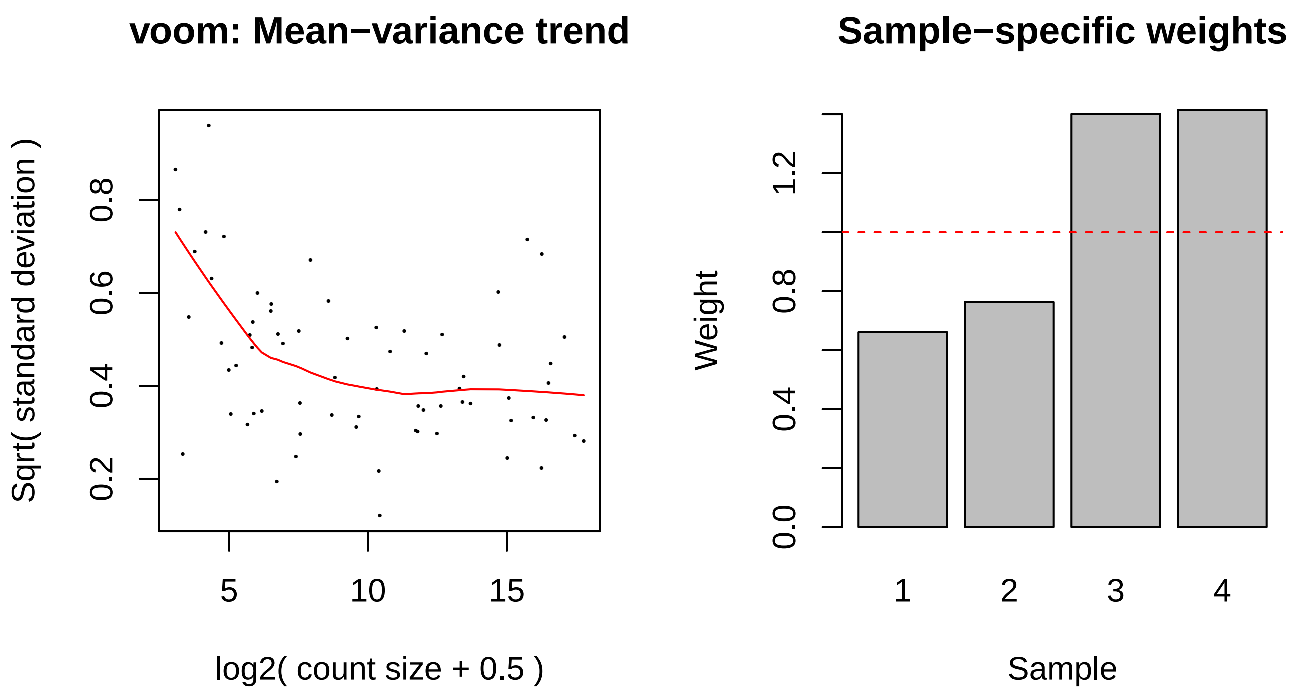

Figure S3. Voom mean-variance trend in the sequins long-read data where points represent genes (left-hand panel), and sample-specific weights obtained from the *voomWithQualityWeights* function (right-hand panel). Even though there were only 69 genes present in the dataset (as opposed to tens of thousands in a typical RNA-seq dataset), the mean-variance trend observed for the sequins data was similar to what one would typically observe in short-read RNA-seq data, with a monotonic decreasing trend (i.e. decreasing variation) as gene abundance increases.

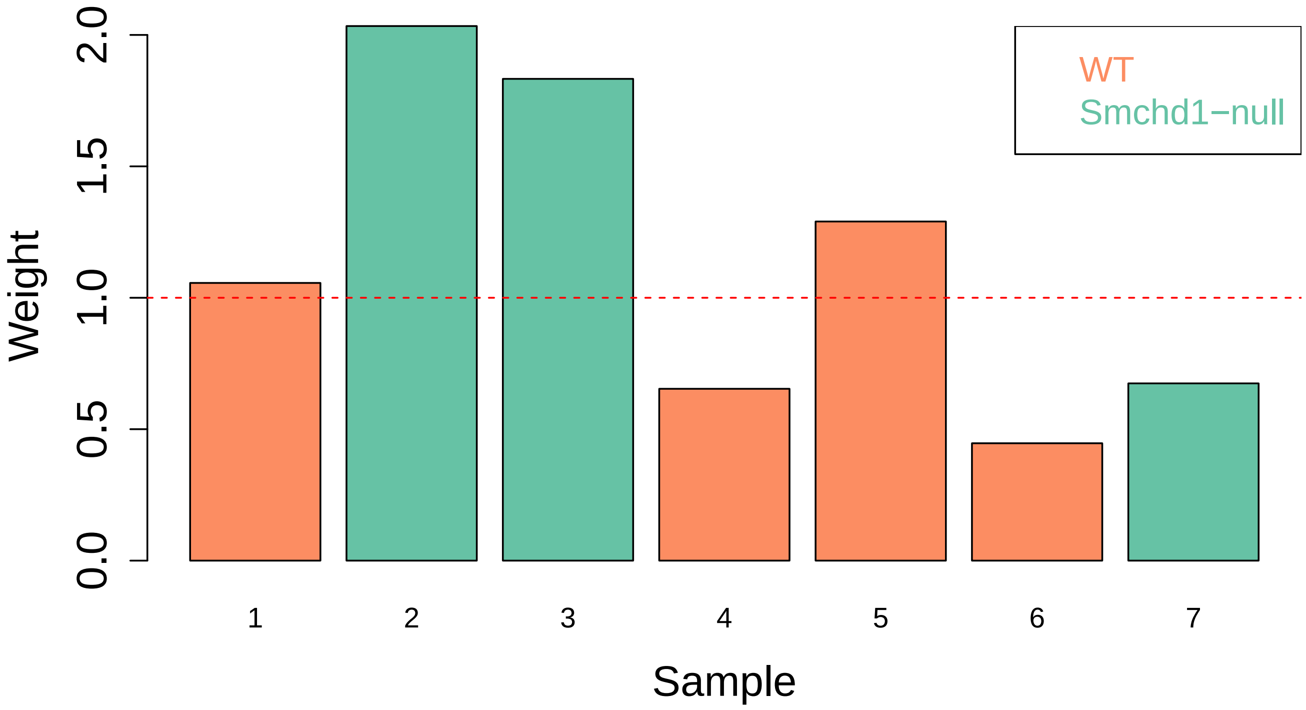

Figure S4. The sample-specific weights of Smchd1-null (green) and WT (orange) NSC long-read samples obtained from the *voomWithQualityWeights* function. Estimated sample weights favoured samples that distinguished groups across dimension 2 of the MDS plot (Figure 2C), giving samples 2 and 3 in the Smchd1-null group weights that are greater than 1, as well as samples 1 and 5 in the WT group.

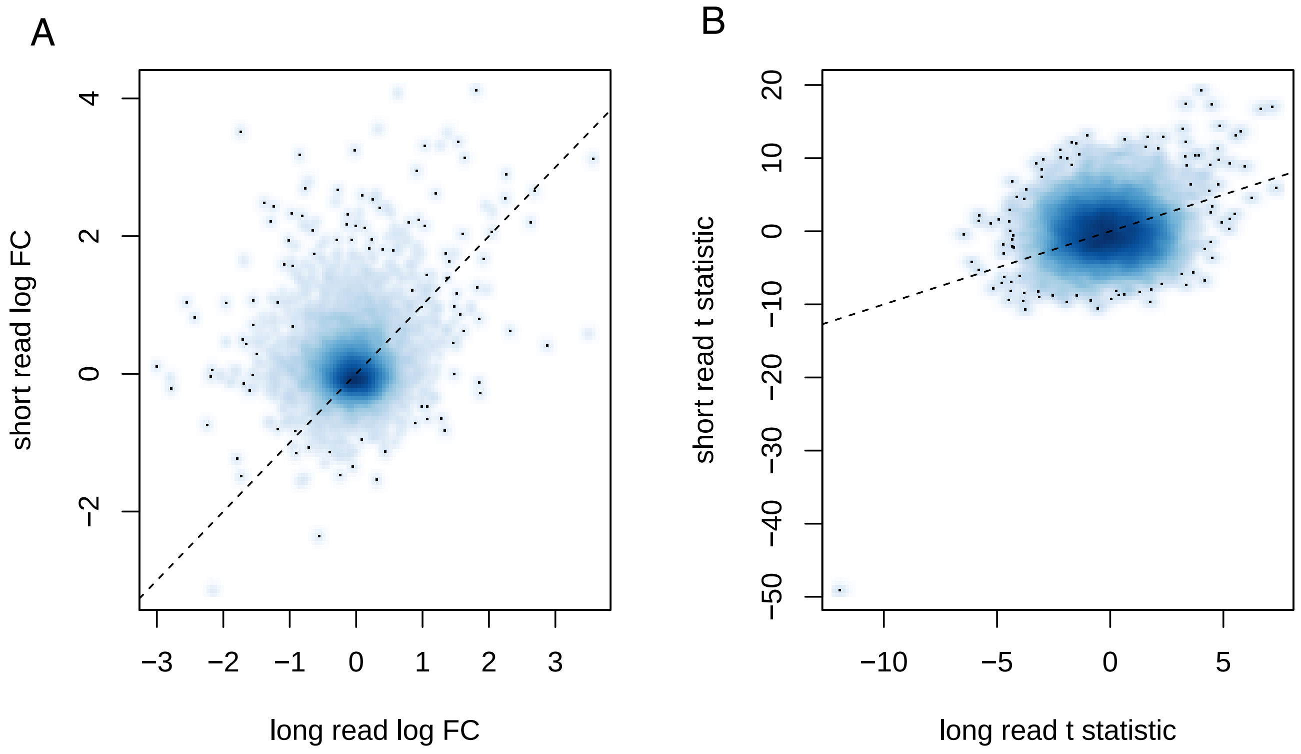

Figure S5. Smoothed scatter plots showing gene-level **(A)** log fold-changes and **(B)** t-statistics from long-read (x-axis) and short-read (y-axis) NSC data, each with a dashed black 'equality' line which has an intercept of 0 and a slope of 1.

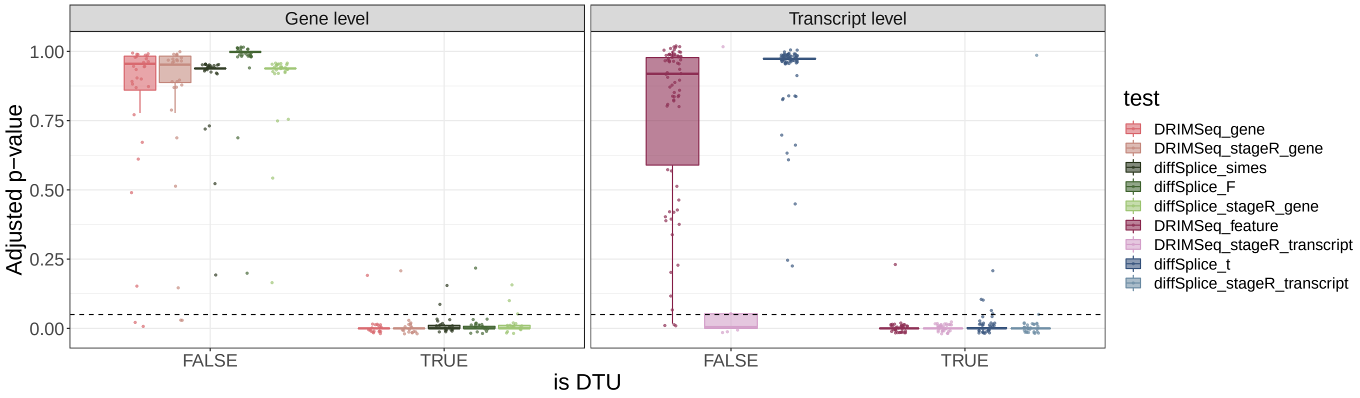

Figure S6. A box plot showing the distribution of adjusted p-values from different tests of DTU for sequins long-read data for known differential (TRUE category) and non-differential (FALSE category) controls. The dashed horizontal line shows the adjusted p-value cutoff of 0.05. Note that transcripts of genes with gene-level raw p-value>0.05 were filtered out in *stageR* and not plotted in the right-hand panel (so for instance, the box for 'diffSplice_stageR_transcript' (light blue) is missing for non-DTU transcripts as they all had a raw p-value>0.05). Non-DTU genes or transcripts which appear under the dashed line in the FALSE category are false discoveries, and DTU genes or transcripts which appear above the dashed line in the TRUE category are false negatives.

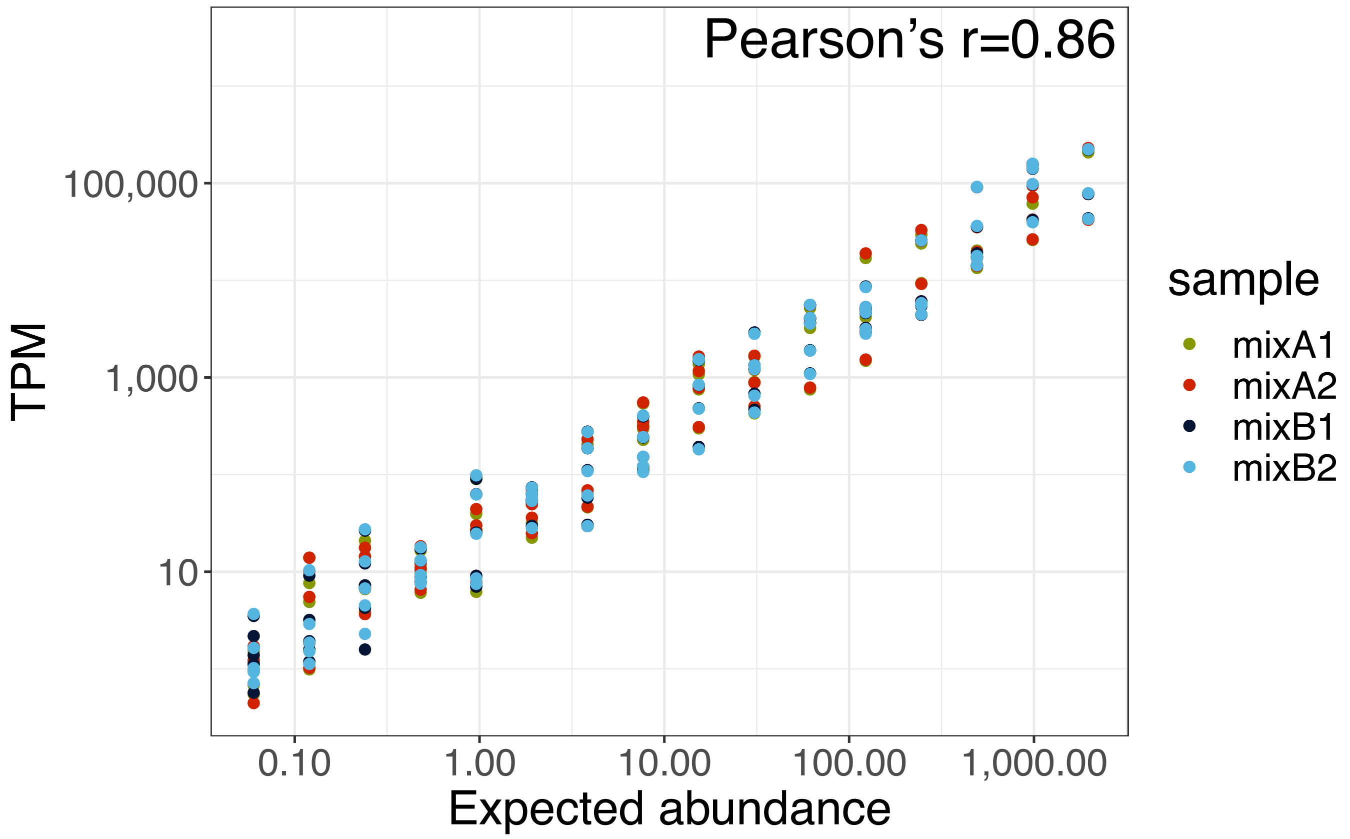

Figure S7. The observed short-read transcript TPM value were highly correlated with expected transcript abundance of each isoform from each sequins sample (Pearson correlation=0.86).

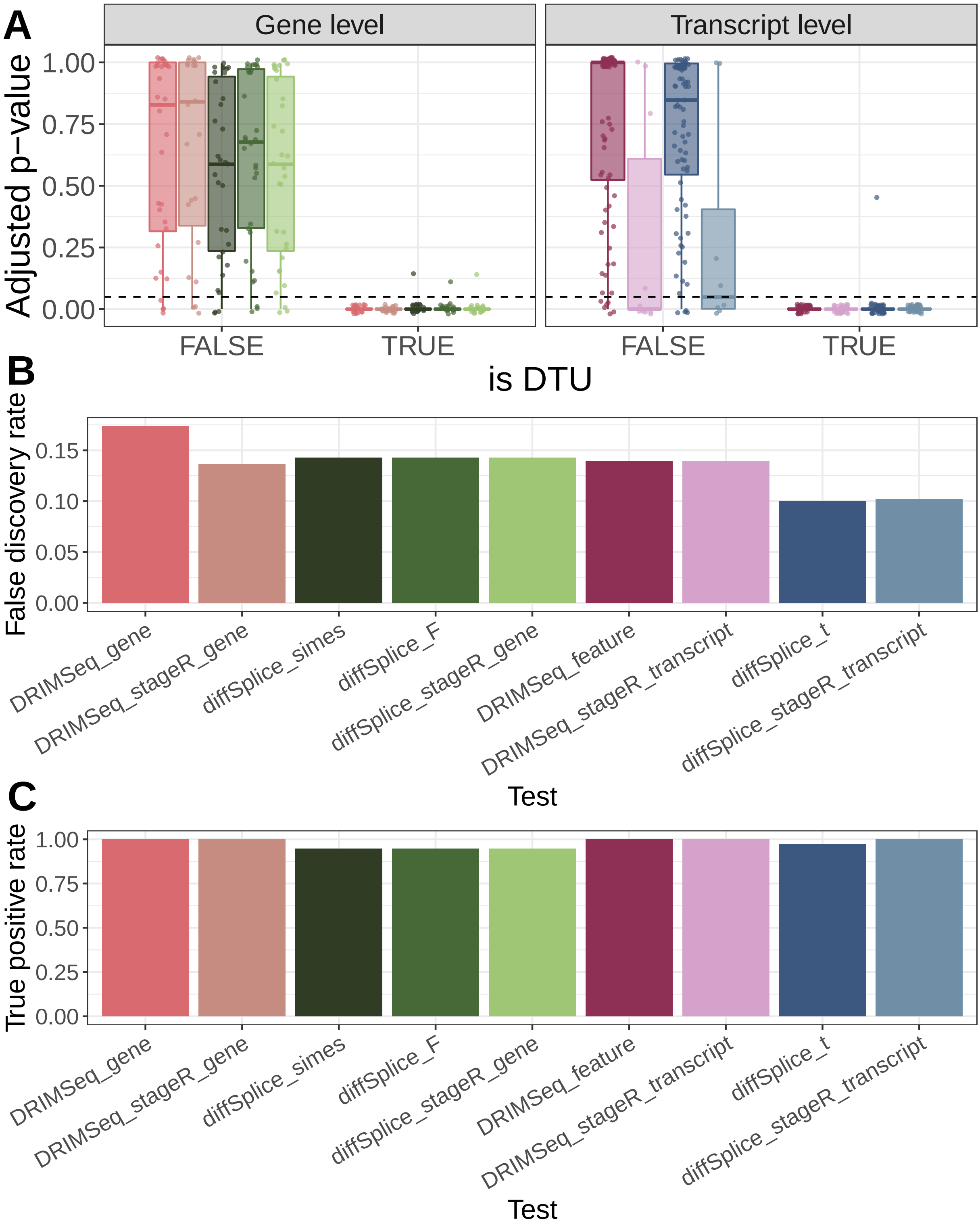

Figure S8. **(A)** A box plot showing the distribution of adjusted p-values from different tests of DTU (this plot uses the same colour-coding as panel **B** and **C**) for the sequins short-read data for known differential (TRUE category) and non-differential (FALSE category) controls. The dashed horizontal line shows the adjusted p-value cutoff of 0.05. Note that transcripts of genes with gene-level raw p-value>0.05 were filtered out from the *stageR* results and are not plotted in the right-hand panel. **(B)** A bar plot showing the false discovery rate (FDR) from different tests of DTU in the sequins short-read data. We expect up to 5% of false discoveries by applying at adjusted p-value cutoff of 0.05. However, the FDR of all DTU tests was above the nominal 0.05 level, which means the error rate was not properly controlled. The FDR was also higher than that obtained for the long-read data (Figure 3E). **(C)** A bar plot showing the true positive rate (TPR) from different tests of DTU in the sequins short-read data.

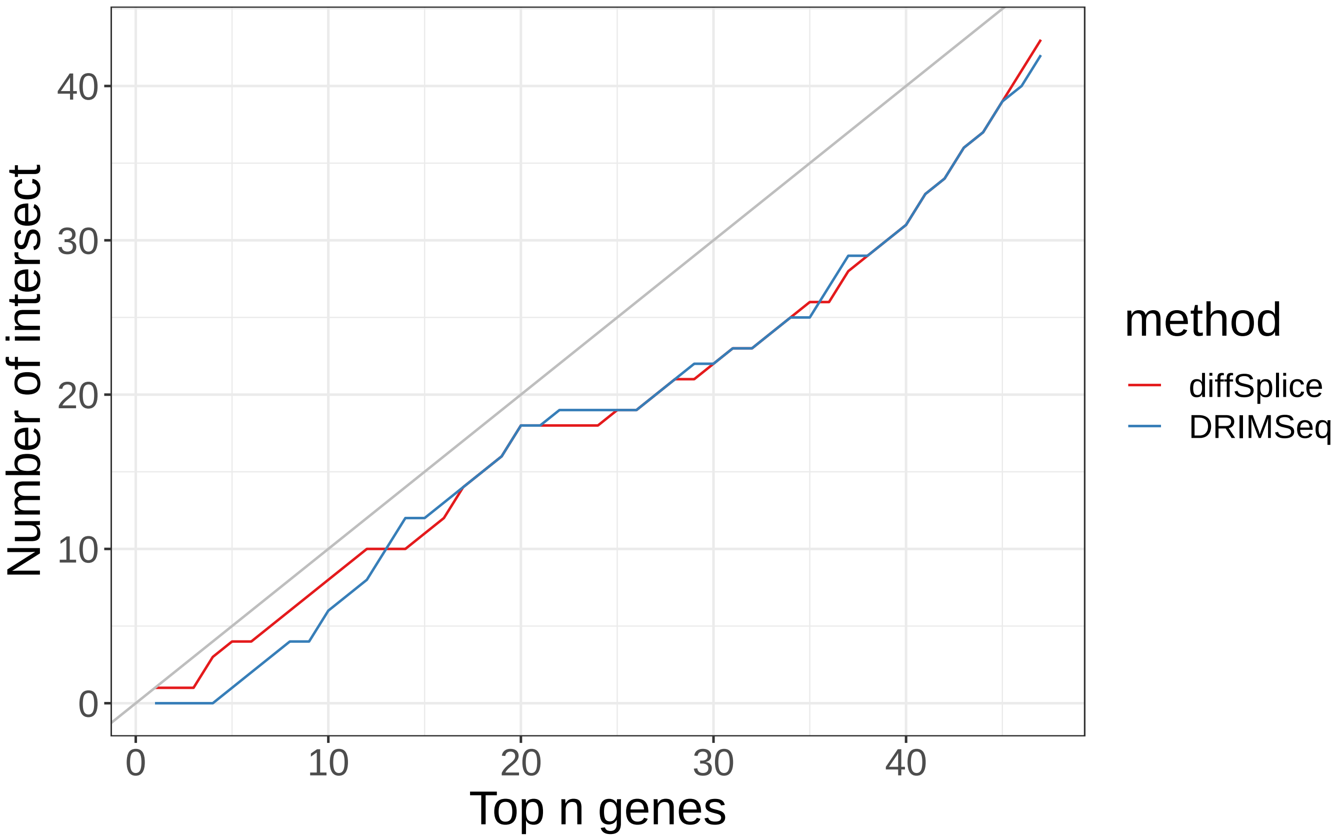

Figure S9. A line chart showing the number of common top n most significant DTU genes between the sequins long- and short-read datasets identified by *diffSplice* (red) and *DRIMSeq* (blue) using a gene-level test. The grey diagonal shows the ideal case where the order of the significance of DTU genes in long- and short-read data are the same. For both *DRIMSeq* and *diffSplice*, in the top 19 genes, which is the number of true DTU genes in these datasets, there were 16 genes in common.

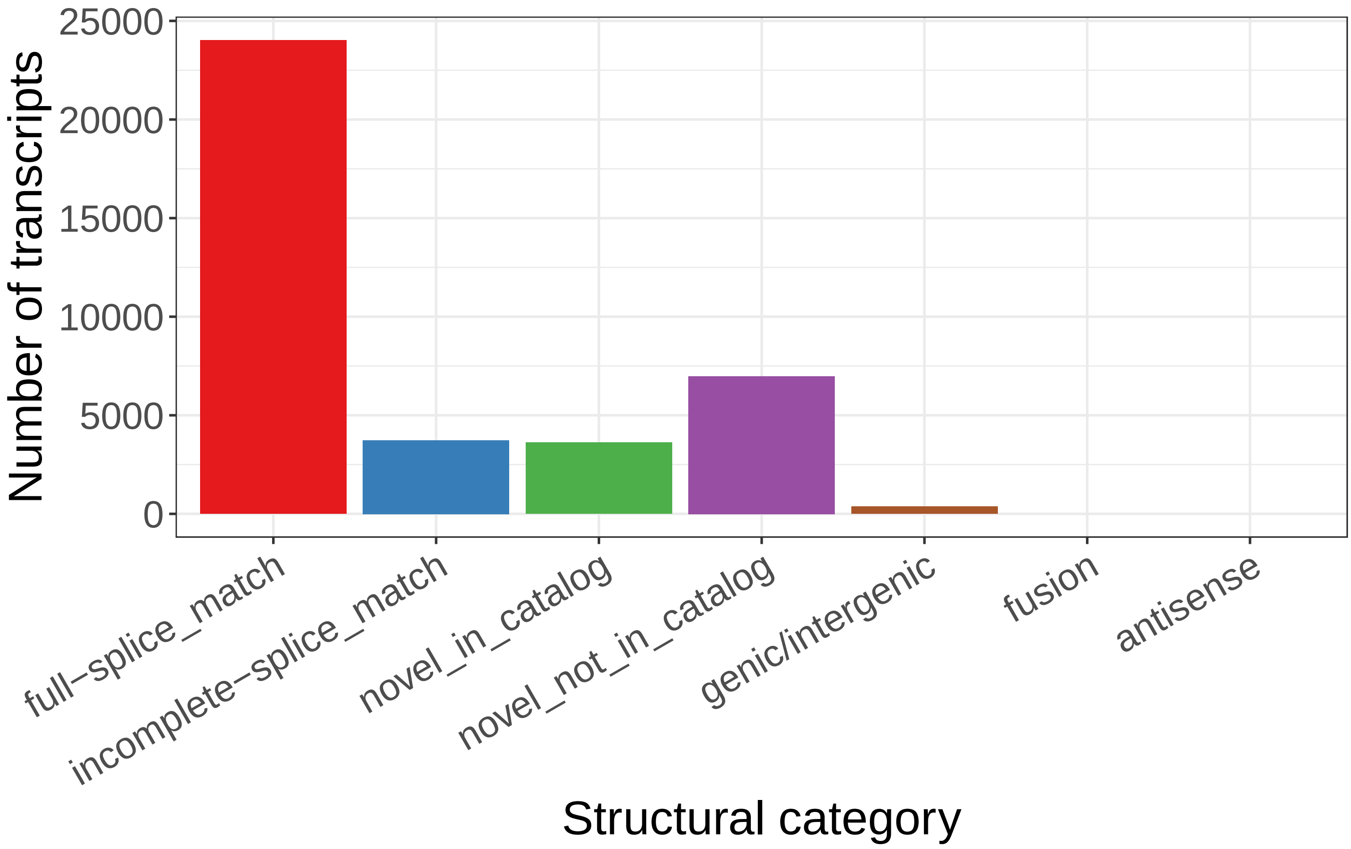

Figure S10. A barplot showing the number of discovered isoforms in each category output by *FLAMES* in the NSC dataset. The *FLAMES* pipeline returned 38,857 unique isoforms from 9,837 genes, of which 38% were classified as novel.

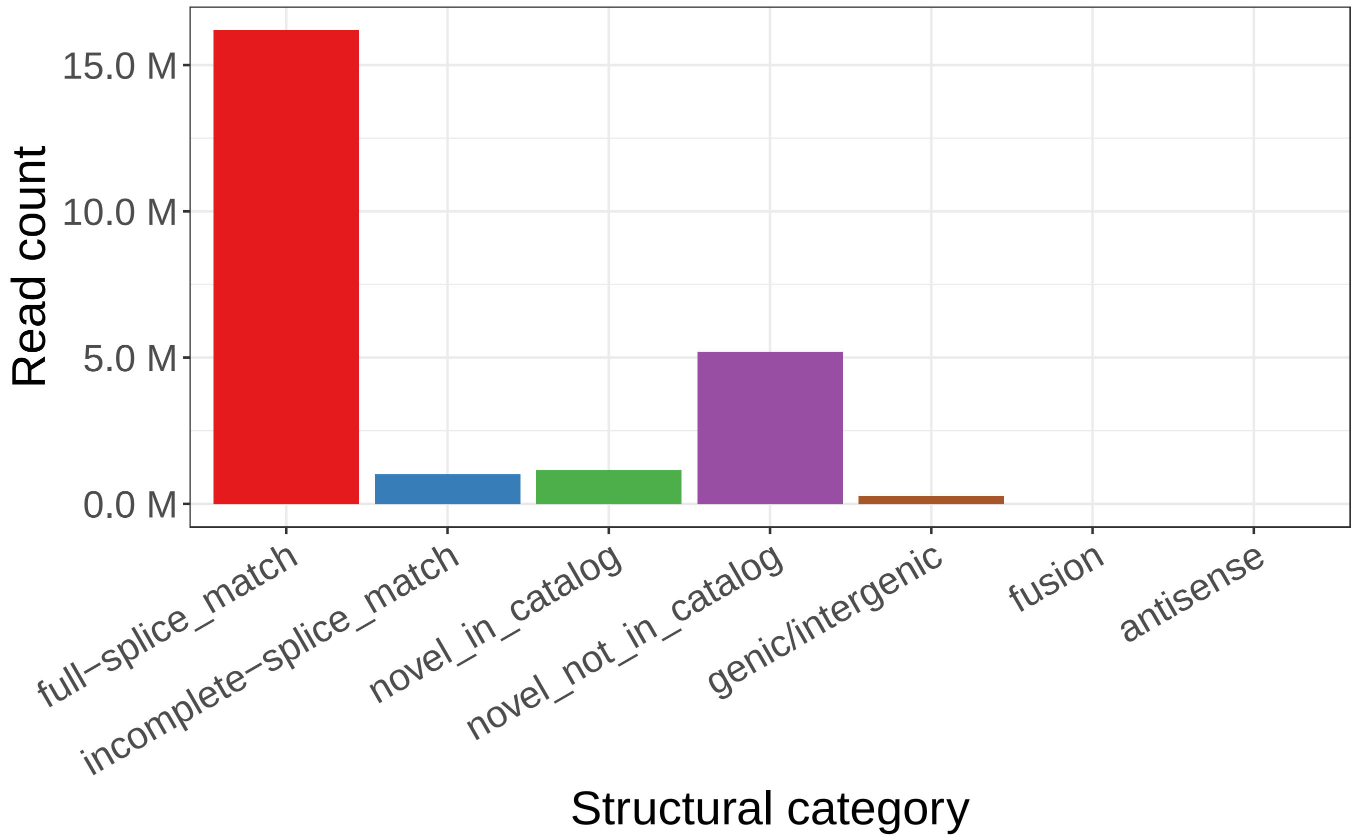

Figure S11. A barplot showing the number of counts from isoforms in each category output by *FLAMES* in the NSC dataset. Of the mapped reads, 32.3% were assigned to novel isoforms, the majority of which were from the 'novel not in catalog' category.

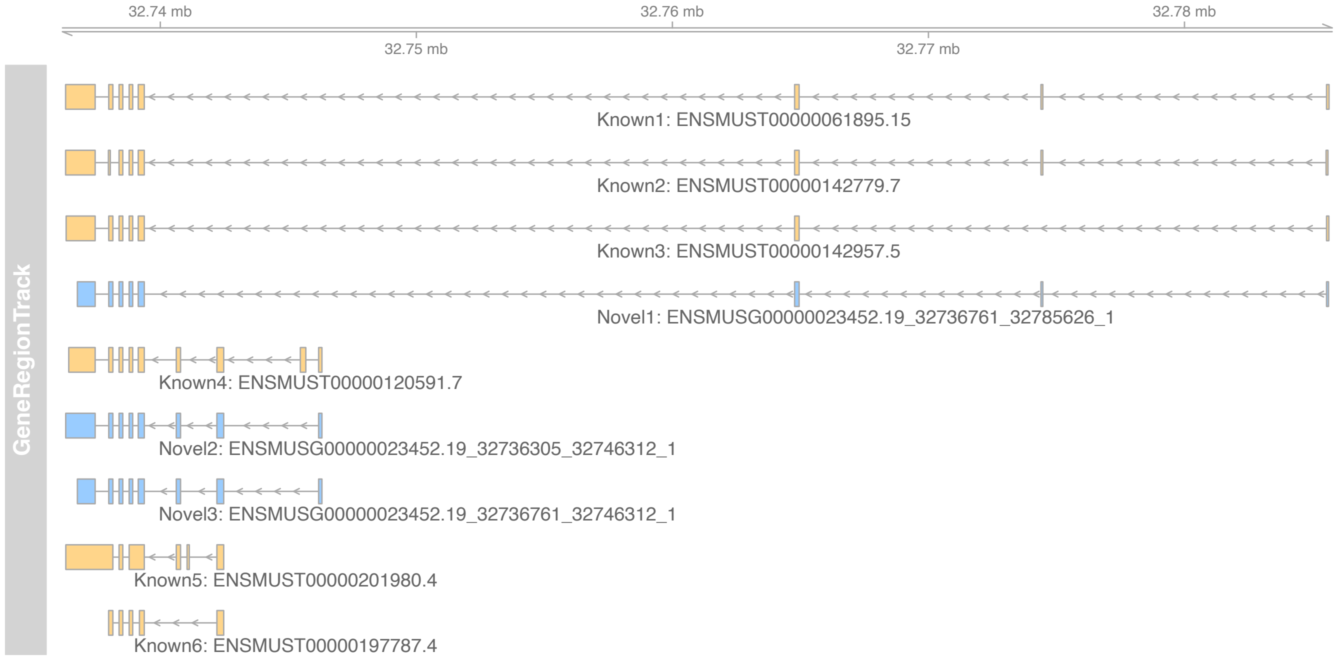

Figure S12. Different isoforms of the gene *Pisd* in the NSC dataset identified by *FLAMES*. Known 1 to 6 (yellow): isoforms present in the dataset which can be found in the reference annotation. Novel 1 to 3 (blue): isoforms which are not present in the reference annotation but were identified in our dataset.

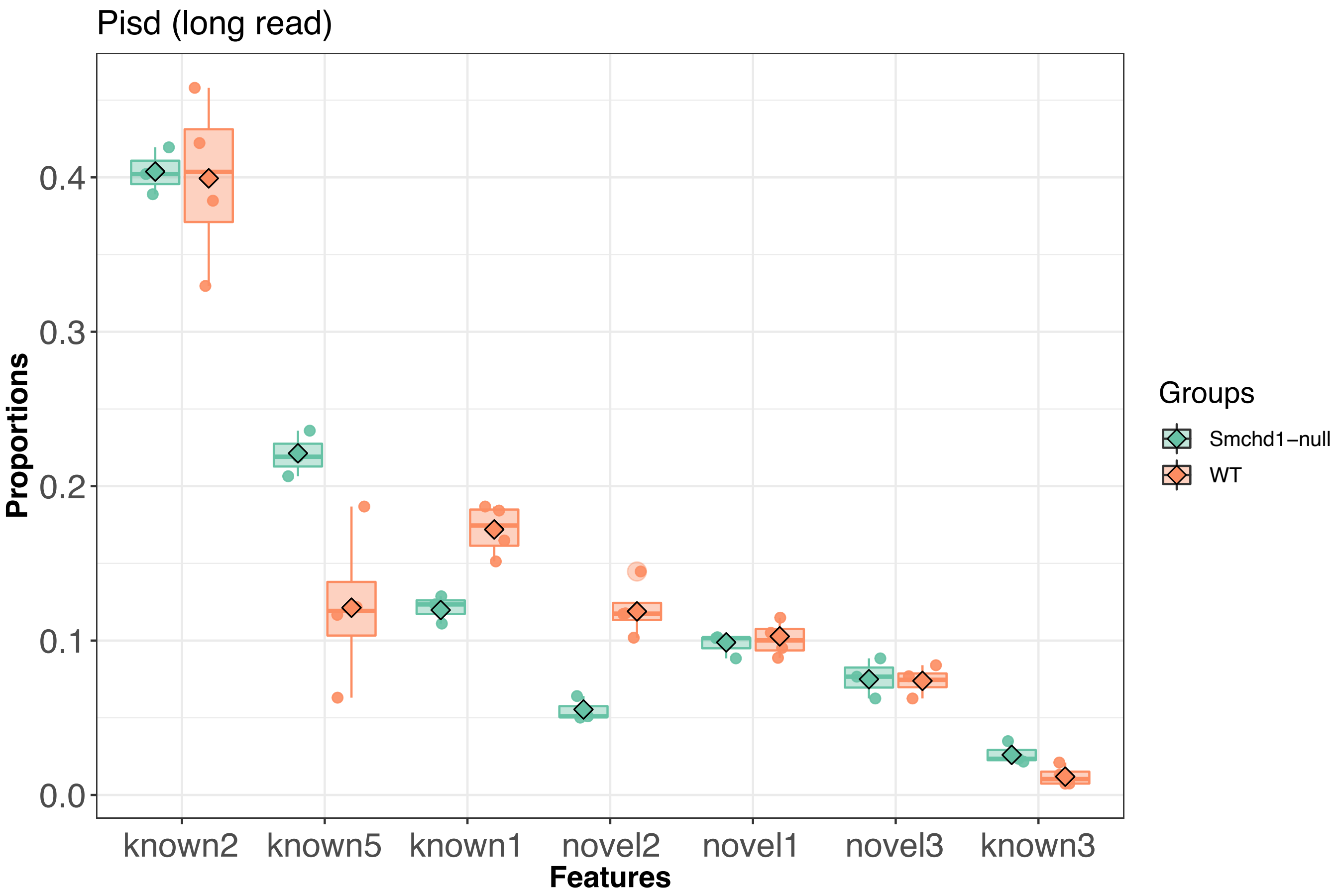

Figure S13. A box plot showing the proportions for each isoform of the gene *Pisd* in the NSC long-read dataset. The diamond marks represent the isoform proportion estimated by *DRIMSeq* in Smchd1-null (green) and WT (orange) samples. Transcript *ENSMUST00000201980.4* ("Known5") and *ENSMUSG00000023452.19_32736305_32746312_1* ("Novel2") in *Pisd* was identified by *DRIMSeq* to have differential usage between the two groups.

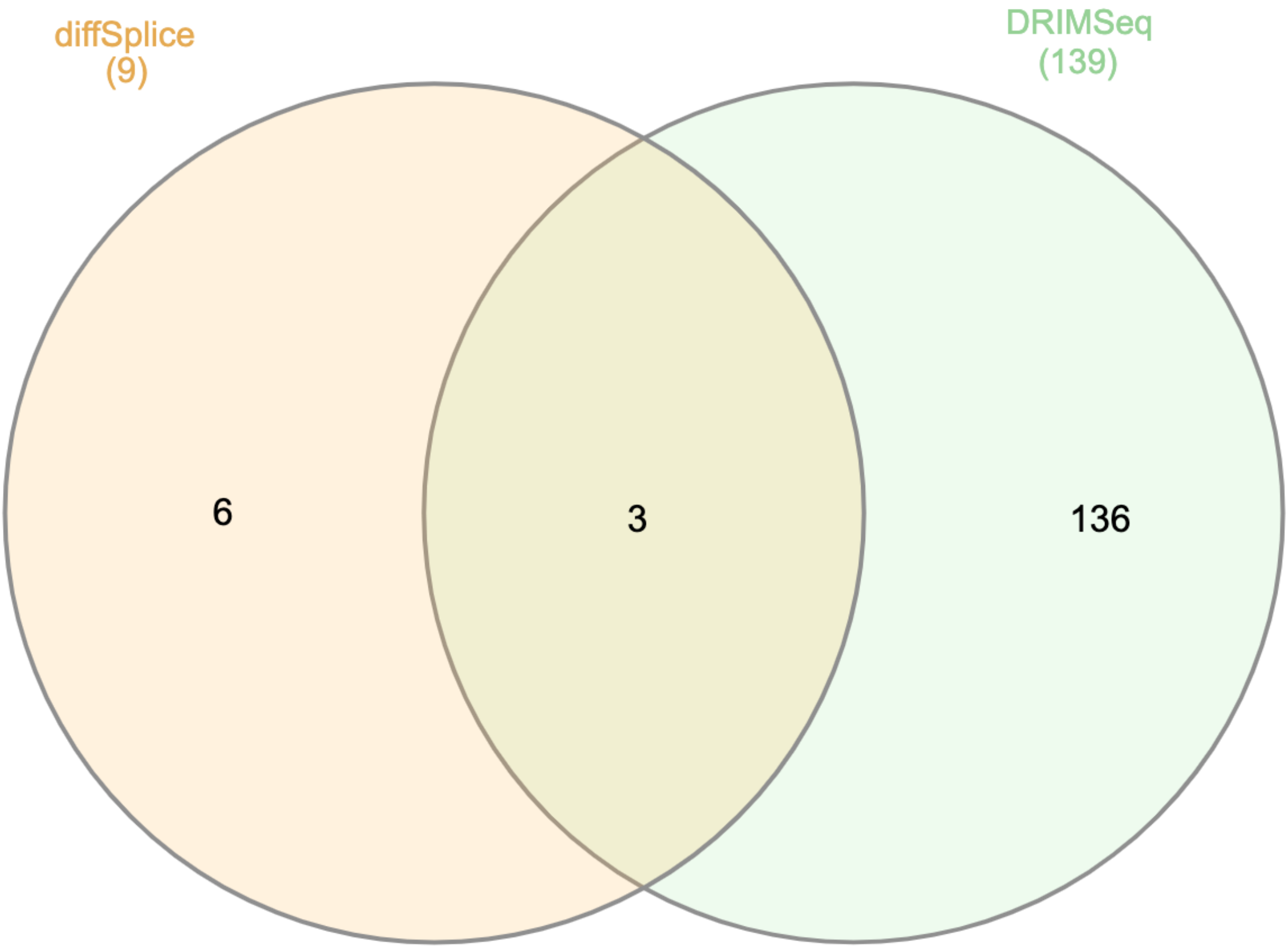

Figure S14. A venn diagram showing the number of DTU genes identified by *diffSplice* (orange) and *DRIMSeq* (green) in the NSC short-read dataset. There were 3 DTU genes (*Pisd*, *Cyth2* and *Pabpn1*) discovered in common by both methods.

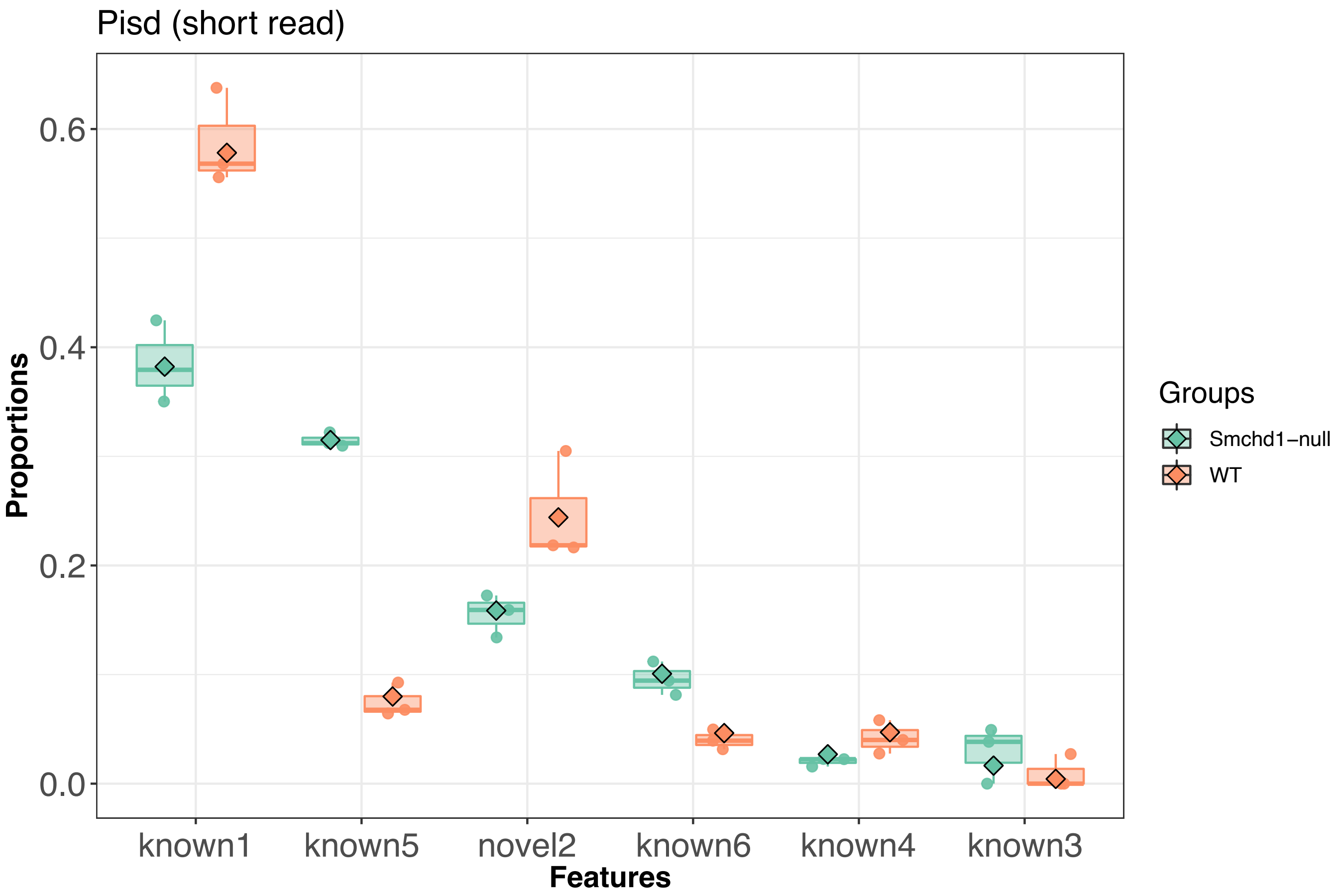

Figure S15. A box plot showing the proportions for each isoform of the gene *Pisd* in the NSC short-read dataset. The diamond marks represent the isoform proportion estimated by *DRIMSeq* in Smchd1-null (green) and WT (orange) samples. Transcript *ENSMUST00000201980.4* (”Known5”) in *Pisd* was identified by both *DRIMSeq* and *diffSplice* to have a higher usage in *Smchd1*-null samples than in WT samples.
